# A case of dyskinesia after levetiracetam administration

**DOI:** 10.1186/s12883-019-1519-8

**Published:** 2019-11-18

**Authors:** Soo Hwan Yim, Yun Ho Choi, Kyoung Heo, Kyoo ho Cho

**Affiliations:** Department of Neurology, Epilepsy Research Institute, Yonsei University College of Medicine, 50-1 Yonsei-ro, Seodaemun-gu, Seoul, Korea

**Keywords:** Levetiracetam, Drug-induced chorea, Dyskinesia, Video-EEG

## Abstract

**Background:**

Antiepileptic drug (AED) induced dyskinesia is an unusual manifestation in the medical field. In the previous case reports describing first generation-AED related involuntary movements, the authors suggested that a plausible cause is pharmacokinetic interactions between two or more AEDs. To date, development of dyskinesia after levetiracetam (LEV) has not been reported.

**Case presentation:**

A 28-year-old woman with a history of brain metastasis from spinal cord glioblastoma presented with several generalized tonic-clonic seizures without restored consciousness. LEV was administered intravenously. Thereafter no more clinical or electroencephalographic seizures were noted on video-EEG monitoring, while chorea movement was observed in her face and bilateral upper limbs.

**Discussion and conclusions:**

To our knowledge, there is no case report of dyskinesia after administration of LEV. Considering the temporal relationship and absence of ictal video-EEG findings, we suggest that development of choreoathetosis was closely associated with the undesirable effects of LEV. We propose that dopaminergic system dysregulation and genetic susceptibility might underlie this unusual phenomenon after LEV treatment.

## Background

Levetiracetam (LEV) is a second-generation antiepileptic drug that is widely used for a variety of seizure types. Intravenous LEV is also regarded as one of the treatment options in status epilepticus. LEV generally has no seriously harmful adverse effects except for psychiatric problems and has quite limited pharmacokinetic interactions with other antiepileptic drugs (AEDs). Dyskinesia including choreathetosis, tremor, and dystonia often develops after acute exposure to certain drugs such as neuroleptics, antiemetics, or psychostimulants. There have been previous reports about movement disorders including tremor and parkinsonism after exposure to AEDs (i.e., phenytoin, valproate, carbamazepine, gabapentin, and phenytoin combined with lamotrigine) [1].

## Case presentation

A 28-year-old woman visited the emergency room with confused mental status after a few generalized tonic-clonic seizure attacks. She had been treated for spinal cord glioblastoma for 4 years. She had undergone surgical removal of a spinal cord tumour and had been on concomitant chemoradiation therapy, which was terminated 2 weeks before the visit. CSF tap was not performed due to an intracranial space occupying lesion.

Brain MRI performed 3 months before the visit showed extension of the tumour from the brainstem to the upper thoracic spinal cord and another lesion at the right temporal pole.

**Fig. 1:**
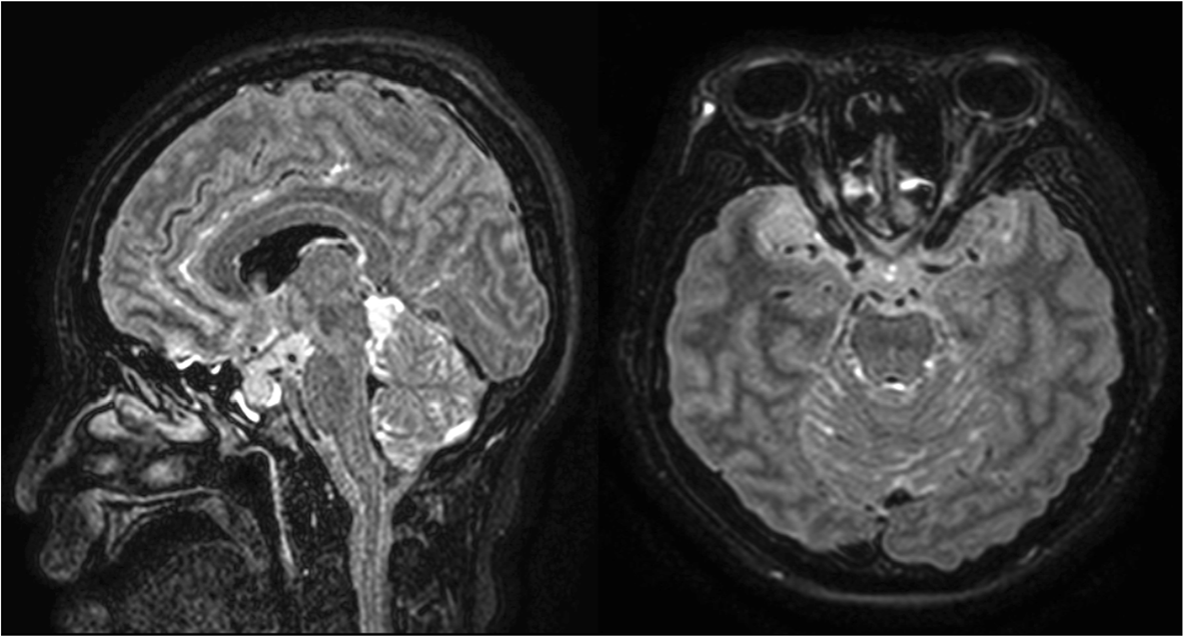
Brain MRI showed extension of glioblastoma of spinal cord to the brainstem and another lesion in the right temporal lobe

**Fig. 2:**
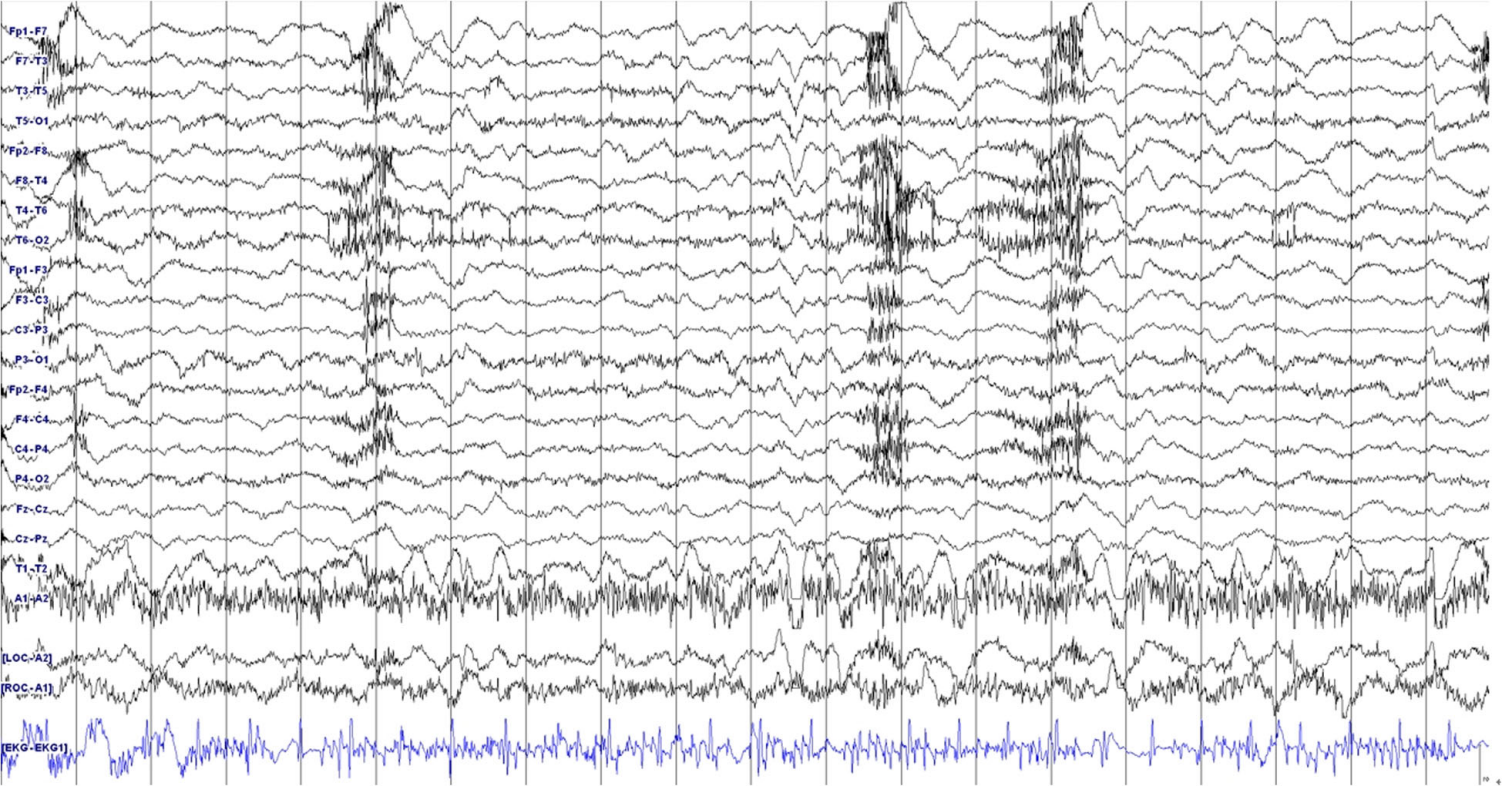
EEG showed no epileptiform discharges during the episode of chorea movement


Figure 1 on presentation, she was drowsy and vital signs were as follows: blood pressure, 125/65 mmHg; body temperature, 36.4 °C; pulse rate, 100 per minute; respiratory rate, 19/min. The emergency medical faculty immediately gave her intravenous LEV 1000 mg; however, there was one more generalised tonic-clonic seizure without mental recovery. Video-EEG monitoring was initiated 2 h after the end of LEV loading; at that time, she started to show choreoathetoid movements in her face and bilateral limbs (see Additional file 1), but no epileptiform discharges were noted on concomitant electroencephalography (EEG) as seen in Fig. 2. Laboratory findings including complete blood counts, electrolytes, blood urea nitrogen, creatinine, and arterial ammonia levels were normal. Review of her medical history revealed that she had been treated with solifenacin, esomeprazole, baclofen, and escitalopram for more than 3 months. She was not on concomitant drugs such as dopamine receptor blockers or antipsychotic agents. Considering the temporal relationship between the onset of involuntary movements and administration of LEV, further treatment with LEV was withheld. Chorea movement continued for about 30 min. She could communicate with her caregiver and physician until the end. After administration of 4 mg of lorazepam and 1200 mg of phenytoin, the involuntary movements ceased gradually. The next day her mental status gradually improved and no more involuntary movements were observed.

**Additional file 1.** Choreoathetoid movements of the patient’s face and bilateral limbs.


## Discussion and conclusions

We judged that the initial seizures were closely related to the patient’s brain lesion (right temporal lobe). Although the drug was not tested by rechallenge, the gradual improvement of chorea and no recurrence after discontinuation of LEV suggests that the patient’s chorea might be attributed to the effect of LEV. Also, underlying metabolic causes related to chorea could be excluded by laboratory findings.

For a seizure originating from mesial temporal lobe and not propagating to lateral structures, the EEG could be negative. However, she was able to communicate while showing the movements, unlike mesial temporal lobe seizures in which consciousness is usually impaired. Moreover, a majority of automatism seizures lasts for less than 1–2 min. Even though we suppose that she was in a complex partial status epilepticus (CPSE), her ongoing movement did not accompany any epileptiform discharges nor clinically evolving features for a relatively long time during video-EEG monitoring, which does not meet the commonly used diagnostic criteria for CPSE. Use of benzodiazepines can alleviate or terminate not only epileptic seizures but also involuntary movements such as tremor, chorea, and dystonia. However, a possibility remains that her symptom was a mesial temporal lobe seizure with oromandibular and manual automatisms because cessation of the movement was observed after administration of other AEDs.

Most cases of anticonvulsant-induced chorea dyskinesia have been reported in association with first-generation AEDs [2]. A possible explanation includes pharmacokinetic interaction with other AEDs or concurrently administered medications, resulting in an additive or synergistic effect on the central dopaminergic pathway [2]. In our case, considering that the patient’s concomitantly administered drugs were in stable use and not generally associated with dopaminergic hyperactivity and furthermore, that LEV has pharmacokinetic drug interactions with them, we supposed that drug-to-drug interactions was not a possible mechanism.

Although the pharmacodynamic effect of LEV is not fully elucidated, inhibition of synaptic vesicle proteins including dopaminergic transmission is speculated to be a probable mechanism for the development of dyskinesia. According to previous reports, LEV showed a therapeutic effect against tardive dyskinesia, which is known to be related to dopaminergic receptor hypersensitivity in long-term neuroleptic users [3]. It was also experimentally shown that LEV has a role in stabilizing levodopa-induced dyskinesia in animals with MPTP-induced lesions [4]. These findings demonstrated that LEV could affect the sensitivity of dopaminergic receptors. In our case, it can be postulated that an acute decrease in synaptic activity of D2 receptors after exposure to LEV was involved in dopamine receptor dysregulation and led to paradoxical hypersensitivity. The approximately 2-h delay between introduction of LEV and development of chorea movements supports this assumption.

Helmstaedter et al. [5] showed an association between genetic variation causing decreased dopaminergic activity and psychiatric effects after LEV treatment, which indicates the importance of appropriate and individualized AED therapy based on a dopaminergic pathway genetic profile. Genetic factors might explain the unexpected phenomenon in our case. People carrying genetic variants associated with altered dopaminergic activity could be at high risk for chorea after LEV treatment. To our knowledge, this is the first report of dyskinesia after LEV treatment. As LEV is widely used in the treatment of epilepsy, clinical vigilance is required when patients present with dyskinesia after initiation of LEV treatment. Also, as can be seen from our case, video-EEG monitoring is an important diagnostic method to not only ascertain if an abnormal movement originated from a true epileptic condition, but also to evaluate and make a differential diagnosis among diverse movement phenomena which can be confusing when treating epilepsy patients.

## Data Availability

Because video clip present both the patient and her caregiver, the data is not publicly available. For requirement of full video clip and clinical and EEG data, kindly refer to the corresponding author (Kyoo Ho Cho).

## Abbreviations

AED: Antiepileptic drugs
CPSE: Complex partial status epilepticus
CSF: Cerebrospinal fluid
EEG: Electroencephalography
GBM: Glioblastoma multiforme
LEV: Levetiracetam
MPTP: 1-methyl-4-phenyl-1,2,3,6-tetrahydropyridine
MRI: Magnetic resonance imaging
